# Supplementary material for: Modulation of receptor-like transmembrane kinase 1 nuclear localization by DA1 peptidases in Arabidopsis
Source: Proc Natl Acad Sci U S A. 2022 Sep 26;119(40):e2205757119. doi: 10.1073/pnas.2205757119 (PMC9546594; doi:10.1073/pnas.2205757119)
Supplement: Supplementary File [file pnas.2205757119.sapp.pdf]

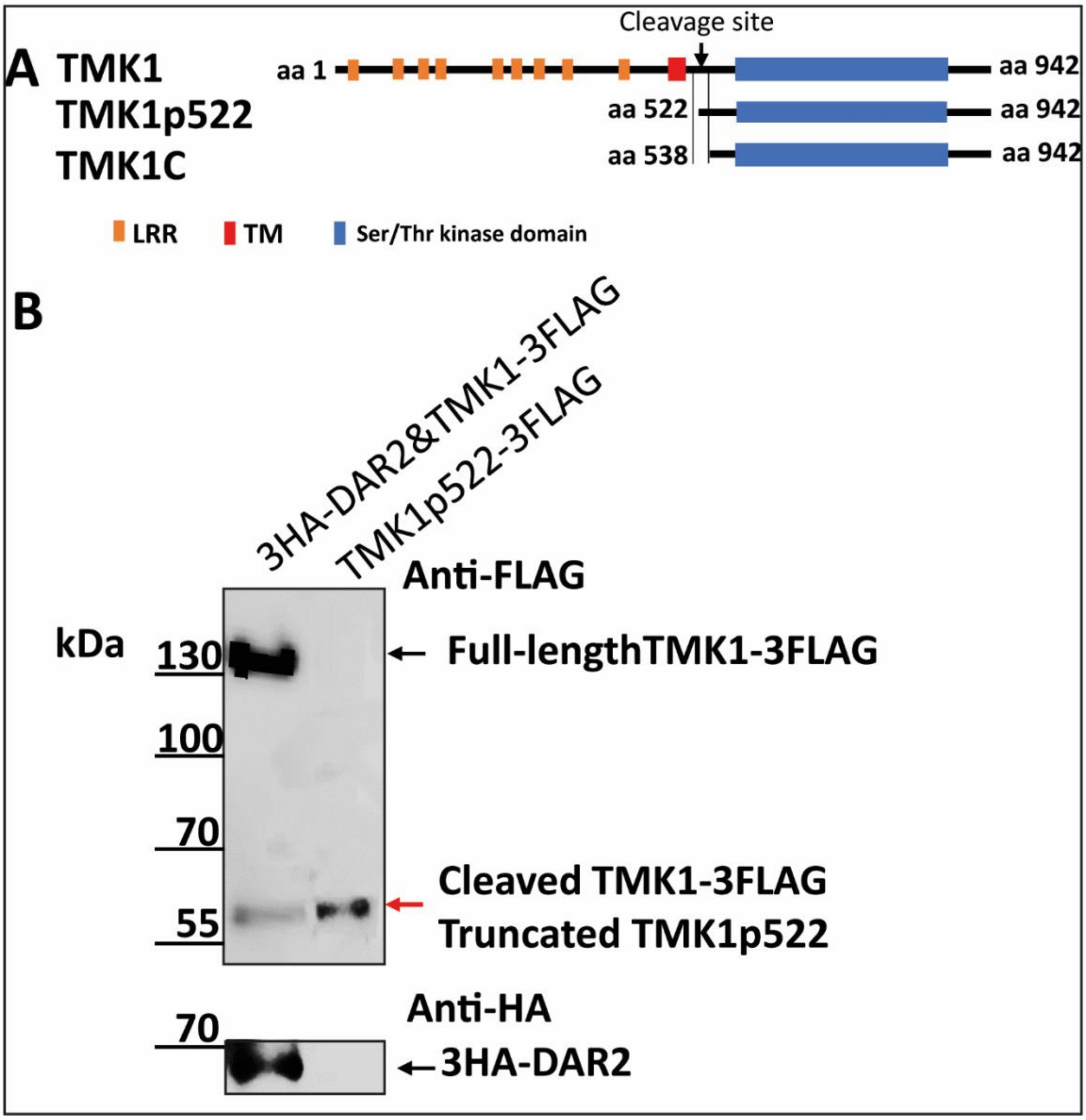

Figure S1. Relative sizes of the TMK1 cleavage band and deletions.

A. The structure of full-length TMK1 and the truncated constructs TMK1p522 and TMK1C. The intracellular kinase domain (blue box) adjacent to the transmembrane domain (red box), and extracellular Leucine Rich Repeats (LRR) (orange boxes) are shown. The start and end amino acid (aa) of each protein is shown. The cleavage site region is located within the 15 aa region shown.

B. Immunoblots showing the relative sizes of cleaved TMK1-3FLAG and the TMK1p522 – 3FLAG deletion construct. The lower panel shows 3HA-DAR2 levels in the protoplast cleavage reaction.

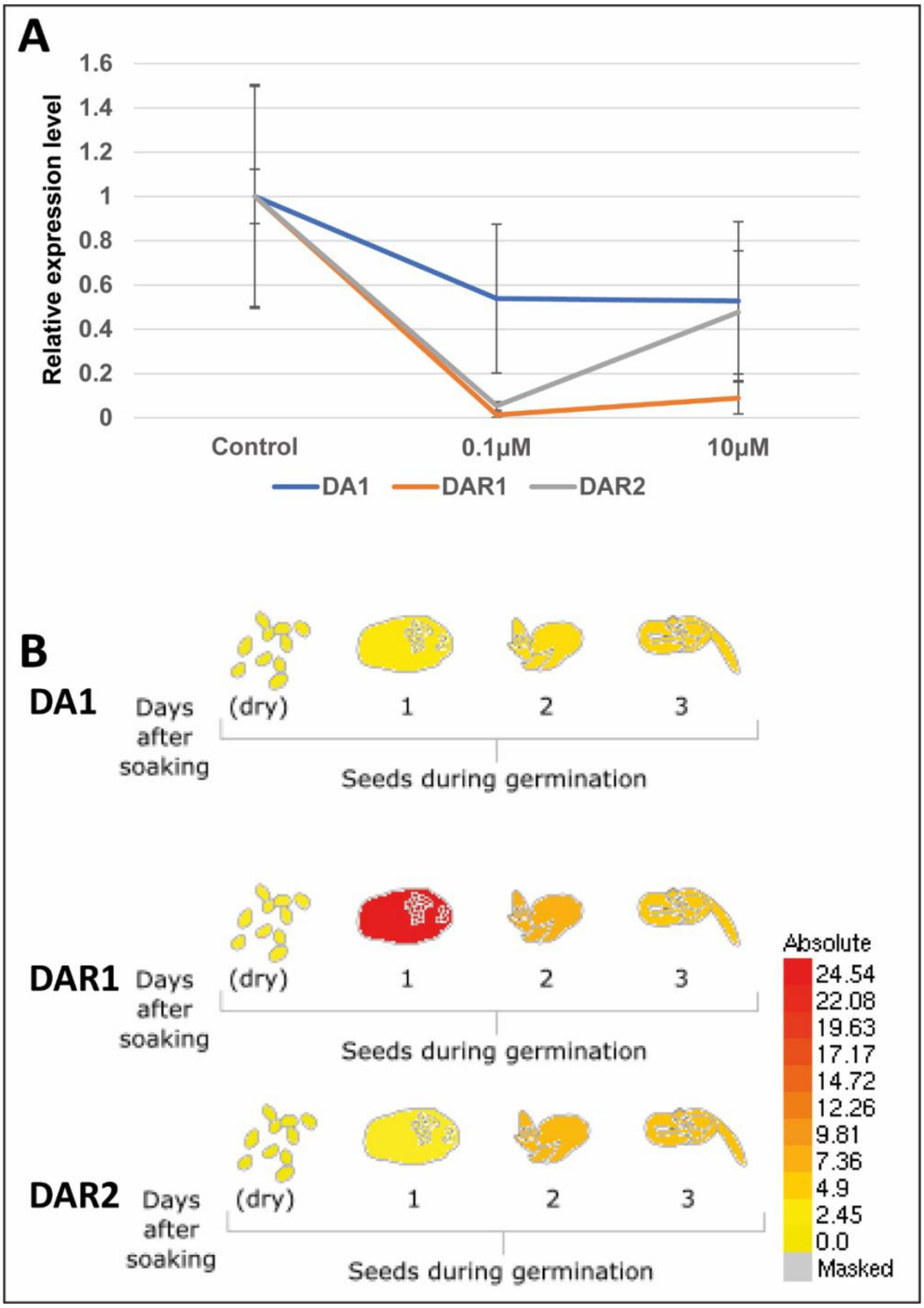

Figure S2. DA1, DAR1 and DAR2 expression level assay.

A. DA1 family peptidases expressional levels are not induced by Auxin. 14 day seedlings were treated by 0.1μM or 10 μM IAA, Ethanol was used as a control. Expression levels are relative to those in control conditions.
B. Expression pattern of *DA1* family peptidases during seed germination（image from https://www.arabidopsis.org/).

Table S1. Primers used

**pENTR/D-TOPO**		
TMK1	Forward	CACC ATGAAGAAAAGAAGAACCTTTCTTCTAT
TMK1p522	Forward	CACC ATGGTAGTGGTGCATCCGC
TMK1C	Forward	CACC ATGATTACAGTTGCGGGTTCAAG
TMK1	Reverse	TCGTCCATCTACTGAAGTGAATG
**In-fusion**		
TMK1p1	Forward	ATGAAGAAAAGAAGAACCTTTCTTCTAT
TMK1p538	Forward	ATGATTACAGTTGCGGGTTCAAG
pENTR/D-TOPO-R	Reverse	GGTGAAGGGGGCGGCCG
		GCCGCCCCCTTCACC
TMK1promoter	Forward	CTTTCTGATCCTTATTTTTTGCT
TMK1promoter	Reverse	TCTTCTTTTCTTCAT AGCTTGAAGAAACAGAGG
TMK1promoter_TMK1C	Reverse	CGCAACTGTAATCAT AGCTTGAAGAAACAGAGG
TMK1(ΔYK)	Forward	CTGTTGGATTACAGTTGCGGGTTC
TMK1(ΔYK)	Reverse	ACTGTAATCCAACAGAAAACTAACAAACC
TMK1(ΔAV)	Forward	GCTCAAATAAGATTACAGTTGCGGG
TMK1(ΔAV)	Reverse	TAATCTTATTTGAGCTCTCACTTCC
TMK1(NAYK)	Forward	AATGCATACAAGATTACAGTTGCGGG
TMK1(NAYK)	Reverse	AATCTTGTATGCATTTGAGCTCTCAC
TMK1(AV-GG)	Forward	CAAATGGAGGAAAGATTACAGTTGCGGG
TMK1(AV-GG)	Reverse	TCTTTCCTCCATTTGAGCTCTCACTTCC
DR5	Forward	GCCGCCCCCTTCACC GAATTCGTCGACGGTATCGC
		CGCAACTGTAATCAT
DR5_TMK1C	Reverse	TGTTATATCTCCTTGGATCGATCC
GoldenGate		
HTB2	Forward	tgtgaagacaa AATG GCGAAGGCAGATAAGAA
		tgtgaagacaa CGAA
HTB2	Reverse	GAACTCGTAAACTTCGTAACC

Table S2. Quantitative RT-PCR premier amplification efficiency

Gene Name	Amplification Efficiency
ACTIN2	2.0823
DA1	2.0160
DAR1	1.9617
DAR2	2.3064
